# Apoptotic Pathway in Intervertebral Disc Degeneration: From Molecular Pathways to Clinical Interventions

**DOI:** 10.3390/diagnostics15121510

**Published:** 2025-06-13

**Authors:** Chae-Gwan Kong, Jong-Beom Park

**Affiliations:** Department of Orthopaedic Surgery, Uijeongbu St. Mary’s Hospital, The Catholic University of Korea College of Medicine, Uijeongbu 11765, Republic of Korea; gongjae@catholic.ac.kr

**Keywords:** intervertebral disc degeneration, apoptosis, mitochondrial dysfunction, inflammation, hyperglycemia, autophagy, ER stress, disc cells

## Abstract

Apoptosis plays a crucial role in the progression of intervertebral disc degeneration (IVDD), a significant cause of chronic low back pain. This review explores disc cell apoptosis’s cellular and molecular mechanisms, focusing on nucleus pulposus, annulus fibrosus, and cartilage endplates cells. Apoptotic pathways—intrinsic (mitochondrial), extrinsic (death receptor-mediated), ER stress-mediated, and autophagy-related—are activated by oxidative stress, inflammation, mechanical load, and metabolic disturbances like hyperglycemia. Diabetes exacerbates disc cell apoptosis through AGE-RAGE signaling and mitochondrial dysfunction. Inflammation further amplifies apoptotic cascades via cytokine signaling and ROS generation. The review also examines emerging therapeutic strategies, including antioxidants (e.g., MitoQ, resveratrol), anti-inflammatory agents (e.g., cytokine inhibitors), autophagy modulators (e.g., rapamycin, metformin), and stem cell and gene therapies. While promising preclinical results exist, challenges such as poor bioavailability and clinical translation remain. Enhanced understanding of apoptosis pathways informs future cellular preservation and matrix integrity treatments. Based on a comprehensive literature search from 2000 to 2025, this narrative review synthesizes current knowledge, identifies knowledge gaps, and discusses translational potential. Our findings support a paradigm shift toward mechanism-based therapies that address the root cause of IVDD rather than symptomatic relief alone.

## 1. Introduction

### 1.1. Scope and Literature Search Strategy

This article is structured as a narrative review aimed at synthesizing current evidence on the role of apoptosis in intervertebral disc degeneration (IVDD) [1,2]. The review elucidates molecular mechanisms, inter-pathway interactions, and translational therapeutic strategies targeting disc cell apoptosis. To ensure comprehensive coverage, we conducted a literature search using PubMed, Scopus, and Web of Science databases up to March 2025. Keywords used included intervertebral disc degeneration, apoptosis, nucleus pulposus (NP), annulus fibrosus (AF), cartilaginous endplates (CEPs), oxidative stress, hyperglycemia, inflammation, autophagy, and regenerative therapies. Only English-language, peer-reviewed studies included original articles, systematic reviews, and meta-analyses. Both in vitro and in vivo experimental studies were reviewed to capture a comprehensive biological perspective. While this review does not follow PRISMA or other systematic frameworks, efforts were made to present an evidence-based synthesis relevant to clinical translation.

### 1.2. Apoptosis in IVDD

Apoptosis in IVDD is mediated by multiple interconnected pathways, including the intrinsic (mitochondrial), extrinsic (death receptor), endoplasmic reticulum (ER)-stress-induced, and autophagy-associated cascades [3,4,5,6]. Proinflammatory cytokines such as tumor necrosis factor-alpha (TNF-α) and interleukin-1 beta (IL-1β) amplify apoptotic signaling via nuclear factor-kappa B (NF-κB) and mitogen-activated protein kinase (MAPK) pathways [7,8,9]. Mitochondrial dysfunction contributes to this process by accumulating reactive oxygen species (ROS), losing membrane potential, and activating caspase. Metabolic disorders, particularly diabetes mellitus, exacerbate disc cell apoptosis by generating excess ROS, inducing mitochondrial damage, and activating the receptor for advanced glycation end-product (RAGE)-mediated pathways through AGEs [10,11]. Simultaneously, the loss of notochordal cells—usually a source of protective growth factors like transforming growth factor-beta (TGF-β) and insulin-like growth factor-1 (IGF-1)—reduces the regenerative potential of the NP.

Recent studies have highlighted additional contributors to apoptosis, including epigenetic dysregulation, impaired autophagy, and microRNA interference. Importantly, apoptosis may occur before visible structural degeneration, reinforcing the value of early intervention [12]. Despite encouraging results in preclinical models, clinical translation remains limited due to the challenging disc microenvironment characterized by hypoxia, acidosis, avascularity, and mechanical stress, which hinders effective drug delivery and therapeutic action [12,13].

Current therapeutic strategies under investigation include cytokine inhibition (e.g., TNF-α and IL-1β blockers), growth factor delivery (e.g., bone morphogenic protein (BMP)-7, TGF-β), metabolic modulation (e.g., antioxidants, insulin therapy), gene therapy, and mesenchymal stem cell (MSC)-based regeneration [14,15,16,17,18,19]. However, significant hurdles remain in optimizing delivery systems, achieving tissue-specific targeting, and minimizing off-target effects.

This review summarizes the principal apoptotic pathways, key risk factors such as inflammation and hyperglycemia, and emerging treatment strategies to preserve disc cell viability and matrix integrity. Advancing our understanding of these mechanisms may facilitate the development of effective disease-modifying therapies for IVDD.

## 2. Anatomy and Cellular Composition of the IVD

The IVD is a fibrocartilaginous structure between vertebral bodies that allows spinal flexibility and distributes axial loads. It consists of three regions: the NP, AF, and CEPs, each with distinct cellular and extracellular matrix (ECM) characteristics essential for disc homeostasis [20,21].

### 2.1. NP Cells

The NP occupies the key portion of the disc and is responsible for maintaining disc hydration and resisting compressive loading. The NP contains a gelatinous ECM primarily composed of proteoglycans (especially aggrecan), glycosaminoglycans (GAGs), and type II collagen, which confer high water content and viscoelasticity to the tissue [21,22,23,24]. 

Notochordal (NC) cells, predominant in early life, are large and vacuolated, secreting anabolic and anti-inflammatory factors such as TGF-β, IGF-1, connective tissue growth factor (CTGF), vascular endothelial growth factor (VEGF), and hepatocyte growth factor (HGF) [16,22,25]. They maintain disc homeostasis via ECM synthesis and apoptosis suppression. Their postnatal loss correlates with reduced regenerative capacity and increased degeneration risk. Species retaining NC cells longer show resistance to IVDD [22,26].

After the disappearance of NC cells, chondrocyte-like cells replace NC cells after adolescence. These smaller, round cells produce ECM components but have limited regenerative capacity and are vulnerable to cytokines, mechanical stress, and oxidative damage [27,28]. They express senescence and apoptotic markers (e.g., p16^INK4a, Bax, caspase-3), and catabolic enzymes (e.g., matrix metalloproteinase (MMP), ADAMTS-5) [24,28,29].

During IVDD, NP cells undergo a phenotypic shift toward catabolic and inflammatory profiles. Degeneration-related changes include downregulation of matrix anabolism, increased MMP and ADAMTS activity, apoptosis, senescence, and impaired autophagy—all contributing to ECM breakdown and disc dehydration [1,20,28,29].

### 2.2. AF Cells

The AF encases the NP and consists of concentric lamellae of collagen fibers arranged in alternating oblique orientations. This architecture provides tensile strength, flexion resistance, and NP containment under mechanical load [21,24]. Fibroblast-like cells aligned with collagen fibers synthesize type I collagen and respond to mechanical stimuli via integrin–FAK–MAPK. Under stress, they increase MMP and cytokine production, weakening the AF [28,29,30].

Regarding regional variations, Inner AF resembles NP with more type II collagen and chondrocyte-like cells. Outer AF is fibrous, rich in type I collagen, and contains elongated fibroblast-like cells, which are key for resisting torsional and bending forces. This regional specialization is essential for the disc to withstand complex multidirectional mechanical loads [21,24].

### 2.3. CEPs Cells

CEPs are thin layers of hyaline cartilage that form the interface between the IVD and the adjacent vertebral bodies. They function as semi-permeable membranes, facilitating the exchange of nutrients and metabolites between the avascular disc and the vertebral capillaries [21,24]. CEPs cells are chondrocyte-like cells embedded within an ECM primarily composed of type II collagen, aggrecan, and hyaluronic acid [24].

Chondrocyte-like CEPs cells produce type II collagen and aggrecan and regulate solute transport. Their function is modulated by mechanical load and inflammation. Degenerative changes include calcification, fibrosis, and thinning, which hinder nutrient diffusion and exacerbate NP degeneration through increased oxidative stress and hypoxia-induced apoptosis [28,30]. CEPs dysfunction exacerbates NP degeneration by reducing nutrient availability, increasing cellular stress, and promoting hypoxia-induced apoptosis in the deeper regions of the disc [28,29].

## 3. Notochordal Cells: Characteristics, Functions, and Role in IVDD

### 3.1. Origin and Development of NC Cells

NC cells arise from the embryonic notochord, which guides early vertebral and neural development via morphogens like Sonic Hedgehog. While vertebral bodies mostly replace the notochord during development, residual cells persist in the NP during early life. In humans, NC cells are typically present at birth and gradually disappear between 10 and 20, with near-complete loss by late adolescence [22,23,24].

### 3.2. Morphology and Cellular Features

NC cells are large, vacuolated cells found in early development. They secrete growth factors—TGF-β, IGF-1, CTGF, HGF—and Wnt inhibitors that inhibit apoptosis and maintain ECM homeostasis [22,23,24]. Their loss is considered an early contributor to IVDD, but their precise role remains under investigation [16,22].

### 3.3. Functional Role in Disc Homeostasis

During early life, NC cells serve as essential regulators of NP homeostasis. Their secretome, composed of growth factors, cytokine modulators, and metabolic enzymes, exerts anabolic, anti-inflammatory, antioxidant, and antiapoptotic effects. These actions help sustain a protective and regenerative microenvironment, promoting disc cell survival, ECM integrity, and long-term disc function [16,22,31,32,33].

- ECM regulation: NC cells enhance ECM synthesis by stimulating the production of aggrecan and type II collagen while suppressing catabolic enzymes such as MMP-13 and ADAMTS-5. This dual action helps preserve the disc’s structural integrity and hydration during early development [16,22].
- Anti-inflammation: NC cells suppress inflammation by inhibiting proinflammatory cytokines like TNF-α and IL-1β and by modulating Toll-like receptor (TLR) signaling pathways. This regulation reduces immune cell infiltration and protects disc cells from inflammation-induced degeneration [16,31].
- Anti-apoptosis: By activating prosurvival signaling pathways such as PI3K/Akt, ERK, and STAT3, NC cells prevent apoptosis, promote disc cell proliferation, and enhance cellular resilience to environmental stressors [16,32].
- Antioxidant defense: NC cells upregulate nuclear factor erythroid 2-related factor 2 (Nrf2) and promote mitochondrial integrity, which enhances cellular resistance to oxidative stress. This antioxidant mechanism helps maintain redox balance and protects disc cells from ROS-induced damage [16,17].

Animal studies using NC cell-rich grafts or NC-conditioned media demonstrated that these interventions can restore disc hydration, improve ECM architecture, and reduce degenerative changes, thereby confirming the therapeutic potential of NC cell-based strategies for disc regeneration [16]. 

### 3.4. Clinical Significance of NC Cells’ Loss and Regenerative Strategies

The disappearance of NC cells contributes to matrix degradation and increased vulnerability to catabolic stressors [16,22,24]. Rather than repeating their general functions, this section focuses on downstream effects, including mitochondrial stress and reduced anabolic capacity [17,20,32]. Therefore, regenerative strategies seek to mimic or restore NC cells’ function, including:

- Differentiation of stem cells into NC cells-like phenotypes.
- Biomaterials incorporating NC cells’ secretome.
- Exosome-based therapies derived from NC cells [14,16].
- Gene editing to reintroduce the NC cells’ markers [16].

These approaches aim to restore the disc microenvironment to a state resembling the NC cells-rich state, thereby slowing or potentially reversing the degenerative cascade [16].

## 4. Pathophysiology of IVDD

### 4.1. Overview of IVDD

IVDD is a complex process that progressively deteriorates disc structure and function. This condition is multifactorial, including genetic, mechanical, and environmental influences [34,35,36]. Age-related degeneration is the most prevalent form and is key in initiating structural disintegration of the NP, AF, and CEPs. Notably, reduced nutrient diffusion due to disc avascularity impairs cell metabolism, contributing to cell senescence and ECM breakdown [37,38,39]. Some studies suggest that hypoxia, lactic acidosis, and oxidative stress promote degenerative cascades in in vitro disc cell models, disrupting disc homeostasis and activating apoptotic signaling in resident cells, thereby accelerating tissue damage and biomechanical failure [40,41,42]. These processes interact synergistically and self-perpetuate, accelerating the degenerative cascade and leading to irreversible structural failure (Table 1).

### 4.2. Loss of Disc Hydration

The NP is the central, gel-like core of the intervertebral disc and plays a critical role in absorbing compressive forces. NP cells maintain high water content by synthesizing proteoglycans, particularly aggrecan, which contain negatively charged GAG side chains that attract and retain water [45].

#### 4.2.1. Proteoglycan Degradation and Water Loss

With aging or pathological stress, matrix-degrading enzymes such as matrix metalloproteinases (MMP-3 and MMP-13) and aggrecanases (ADAMTS-4 and ADAMTS-5) become upregulated, leading to the degradation of aggrecan and other proteoglycans [34,35]. In vivo and ex vivo models confirm that reduced GAG content leads to decreased osmotic pressure, diminished hydration, and reduced disc turgor. These biochemical changes result in visible disc desiccation on MRI and impair the disc’s ability to resist axial compression [34,35,36]. 

#### 4.2.2. Collagen Composition and Fibrosis

Healthy NP tissue contains type II collagen, which provides elasticity and facilitates water retention. A phenotypic shift toward type I collagen, a hallmark of fibrosis, is observed in degenerated discs. Evidence from histological analysis of human degenerated discs supports this collagen transition. This shift reflects a transformation from a cartilaginous to a fibrocartilaginous phenotype, resulting in disc stiffening and reduced shock-absorbing capacity [37,38].

#### 4.2.3. Consequences of Dehydration

The loss of hydration results in reduced disc height, increased intradiscal pressure heterogeneity, and altered stress distribution across the AF and CEPs. These changes predispose the annulus to fissure, as supported by biomechanical studies linking matrix degradation to reduced tensile strength [38]. Mechanical overload from reduced disc height leads to facet joint degeneration and adjacent segment disease.

### 4.3. ECM Degradation

The ECM forms the structural scaffold of the intervertebral disc and is essential for maintaining biomechanical integrity and regulating cell behavior. It comprises proteoglycans, collagen, elastin, and non-collagenous proteins [39].

#### 4.3.1. Imbalance in ECM Turnover

In IVDD, this balance shifts toward catabolism due to the upregulation of MMPs and aggrecanases, particularly ADAMTS enzymes. Animal model studies and human disc tissue analyses have demonstrated that proinflammatory cytokines and mechanical stress induce key enzymes—MMP-1, MMP-3, MMP-13, and ADAMTS-5—[39,40]. 

#### 4.3.2. Suppression of Anabolic Factors

Some studies suggest that the downregulation of anabolic growth factors, including TGF-β, IGF-1, and BMP-7, is associated with advanced IVDD in vitro and in vivo models [39]. These factors are essential for maintaining aggrecan levels, collagen synthesis, and NP cell survival. Reduced signaling through the Smad2/3 and PI3K/Akt pathways contributes to impaired matrix regeneration. Additionally, ECM fragments function as damage-associated molecular patterns (DAMPs), further stimulating catabolic and inflammatory responses [40].

#### 4.3.3. Structural and Functional Outcomes

ECM degradation leads to reduced disc elasticity and eventual mechanical failure. Softening of the NP increases the mechanical load transferred to the AF, rendering it more susceptible to delamination and radial tears [40,41]. Calcification and sclerosis of the CEPs impair nutrient diffusion, thereby reducing glucose and oxygen supply to disc cells and exacerbating degeneration [42].

### 4.4. Inflammation

Chronic, low-grade inflammation is a hallmark of IVDD and contributes to ECM degradation, disc cell death, and nociceptive sensitization. Proinflammatory cytokines such as TNF-α, IL-1β, and IL-6 are significantly elevated in degenerated human discs and have been shown in vitro to promote apoptosis through NF-κB and MAPK signaling [3,11,13,29,30].

#### 4.4.1. Key Inflammatory Mediators

In degenerated discs, proinflammatory cytokines—including TNF-α, IL-1β, IL-6, and IL-17—and chemokines such as monocyte chemoattractant protein-1 (MCP-1) are significantly elevated. Immunohistochemical analysis in human IVDD samples has demonstrated increased levels of these cytokines and their receptors [3,11,13,29,30].

#### 4.4.2. Inflammation-Induced Degeneration

In vitro models have shown that inflammatory mediators suppress anabolic gene expression, increase the generation of ROS, and sensitize cells to apoptosis and senescence [3,11,13,18,29]. Cytokines upregulate death receptors (e.g., Fas and DR5) and mitochondrial pro-apoptotic factors, further promoting disc cell loss [3,13,15]. Cytokine-induced expression of neurotrophic factors, like nerve growth factor (NGF) and brain-derived neurotrophic factor (BDNF), contributes to abnormal nerve ingrowth and pain sensitization, especially at the locations of annular fissures [8,30].

### 4.5. Oxidative Stress

Oxidative stress, marked by excessive ROS accumulation, is a key inducer of apoptosis in IVDD. In vitro studies using human and rat NP cells have shown that exposure to hydrogen peroxide (H_2_O_2_) increases caspase activity and DNA fragmentation [35,36]. Elevated ROS levels—confirmed in both aged human discs and animal models—lead to lipid peroxidation, protein oxidation, mitochondrial dysfunction, and impaired ATP synthesis [1,28,35]. Some studies suggest that antioxidants such as N-acetylcysteine and mitoquinone partially restore redox balance and mitigate apoptosis [4,17,35].

#### 4.5.1. Mitochondrial Damage and Antioxidant Deficiency

ROS promotes mitochondrial membrane depolarization, cytochrome c release, and intrinsic apoptotic signaling [5,17,35]. In degenerated discs, antioxidant enzyme expression is reduced, exacerbating oxidative injury [35,36]. This dual effect—elevated ROS and impaired defense—accelerates disc cell vulnerability to apoptosis [4,17,35].

#### 4.5.2. ROS–Cytokine Synergy

ROS also activates NF-κB and AP-1 pathways, stimulating inflammatory gene expression [3,36]. Proinflammatory cytokines enhance ROS production, creating a feed-forward loop [29,36]. This synergy amplifies apoptosis, ECM degradation, and nociceptive mediator expression [1,3,28,36].

### 4.6. Apoptosis and Necrosis

The loss of intervertebral disc cells through both regulated (apoptosis) and unregulated (necrosis) forms of cell death is a critical driver of IVDD progression and structural deterioration of the disc matrix [43]. These processes result in diminished ECM synthesis, mechanical instability, and exacerbation of inflammation, ultimately accelerating the degenerative cascade.

#### 4.6.1. Apoptosis

Apoptosis in IVDD is predominantly mediated through mitochondrial (intrinsic), death receptor (extrinsic), ER stress-mediated, and autophagy-associated signaling pathways. Increased expression of key pro-apoptotic markers—including Bax, cleaved caspase-3, CHOP, and caspase-12—has been consistently observed in both NP and AF cells isolated from degenerated human discs [3,19,20,28]. These findings are supported by in vitro studies using cultured disc cells and in vivo animal models of disc degeneration [3,19,20]. Apoptotic signaling is further amplified under pathological conditions by proinflammatory cytokines (e.g., TNF-α, IL-1β), mitochondrial dysfunction, and excessive production of ROS [11,20,28,36,46]. Such stimuli promote mitochondrial membrane permeabilization, cytochrome c release, and caspase activation, leading to apoptosis.

#### 4.6.2. Necrosis and Necroptosis

In contrast to apoptosis, necrosis is an unregulated cell death typically resulting from extreme stress or trauma. Recently, necroptosis—an intermediate, programmed form of necrosis—has been identified as a contributor to inflammation-driven disc degeneration. Necroptosis is primarily mediated by receptor-interacting protein kinases RIPK1 and RIPK3 and the execution protein MLKL [45,47]. MLKL disrupts the cell membrane upon activation, releasing damage-associated molecular patterns (DAMPs) that amplify inflammation. Animal models of IVDD have shown that RIPK3 inhibition reduces necroptosis and cytokine expression, suggesting necroptosis as a potential therapeutic target for mitigating inflammation-driven disc degeneration [45]. 

IVDD arises from a complex interplay among biomechanical, biochemical, and molecular factors [1,34]. Loss of hydration, ECM degradation, inflammation, oxidative stress, and cell death interact dynamically to destabilize disc homeostasis and accelerate degeneration [3,20,28,36]. These pathological processes are not isolated; they form interdependent feedback loops reinforcing one another [1,20,31,34]. Understanding these interrelated pathways provides a scientific foundation for developing multimodal, disease-modifying therapies that target both the symptoms and the underlying mechanisms of intervertebral disc degeneration [3,31,38].

## 5. Mechanism of Disc Cell Apoptosis in IVDD

**Figure 1:**
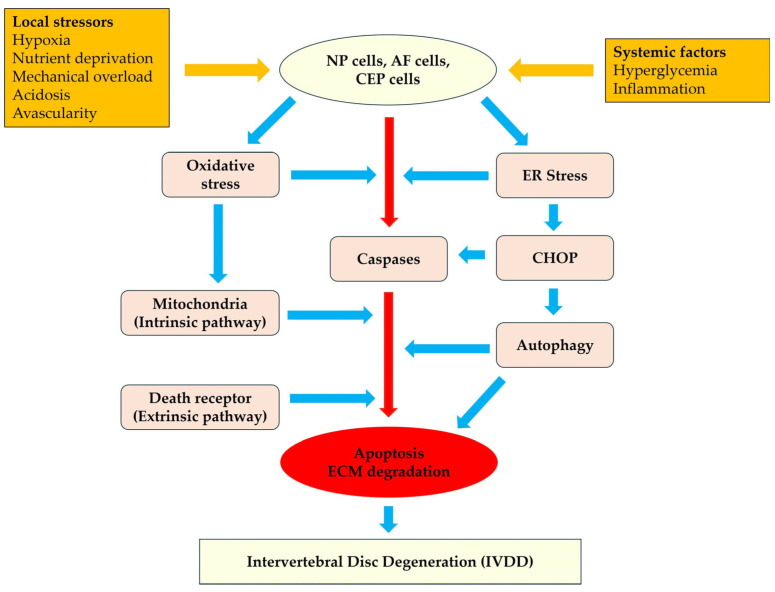
Molecular pathways of apoptosis in intervertebral disc degeneration (IVDD). This diagram illustrates the converging apoptotic pathways in IVD cells, including NP, AF, and CEPs cells, under the influence of both local and systemic stressors. Local stressors such as hypoxia, nutrient deprivation, acidosis, mechanical overload, and avascularity directly activate oxidative stress and endoplasmic reticulum (ER) stress in disc cells. Systemic factors, including hyperglycemia and chronic inflammation, amplify these stress responses. Four primary apoptotic signaling cascades are depicted: Intrinsic (mitochondrial) pathway [3,4,5]: Triggered by oxidative stress, mitochondrial dysfunction, and DNA damage, leading to cytochrome c release, apoptosome formation, and caspa-se-9/3 activation. Extrinsic (death receptor-mediated) pathway [3,9,28,30]: Activated by inflammatory cytokines such as TNF-α and Fas ligand (FasL), engaging receptors (e.g., TNFR1 and Fas initiate caspase-8 activation and interact with the intrinsic pathway through Bid cleavage. ER stress pathway [7,47]: Prolonged ER stress upregulates CHOP, which downregulates Bcl-2 and promotes mitochondrial-mediated apoptosis; this can activate caspase-12 and link to mitochondrial and autophagic pathways. Autophagy-apoptosis crosstalk [10,13,20,48,49]: Autophagy protects against apoptosis by degrading damaged organelles. However, dysregulated autophagy triggers apoptosis under chronic stress through Beclin-1 cleavage and p62 accumulation. All pathways converge on caspase activation, ultimately resulting in apoptosis and degradation of the ECM, which drives structural failure and disc degeneration. Arrows in the figure highlight the feedback and crosstalk between pathways.

Apoptosis is key in IVDD progression, occurring through intrinsic, extrinsic, ER stress-mediated, and autophagy-associated pathways. The intrinsic pathway, activated by mitochondrial damage and oxidative stress, causes cytochrome c release and caspase-9 activation [3,4,5]. The extrinsic pathway, initiated by death receptors such as Fas and TNFR1, activates caspase-8 and intersects with mitochondrial signals via Bid cleavage [3,9,28,30]. The ER stress pathway becomes prominent during disease when unfolded proteins accumulate, upregulating CHOP and activating caspase-12 [7,47]. Autophagy, cytoprotective under physiological conditions, becomes maladaptive under chronic stress, promoting apoptosis through Beclin-1 cleavage [10,13,20,48,49]. These mechanistic insights are supported primarily by in vitro studies using human and animal disc cell cultures and ex vivo disc explant models. Notably, these pathways are interconnected, amplifying apoptotic effects through cross-signaling loops (Figure 1):

- The intrinsic (mitochondrial-mediated) pathway.
- The extrinsic (death-receptor-mediated) pathway.
- The ER-stress-induced pathway.
- Autophagy-associated pathways.

These apoptotic cascades are triggered by oxidative stress, inflammation, mechanical overload, hyperglycemia, and aging. Importantly, these stimuli often converge and interact, amplifying disc cell apoptosis and accelerating degenerative progression.

### 5.1. Intrinsic (Mitochondrial) Pathway

Internal stress signals—including oxidative damage, DNA injury, and mitochondrial dysfunction—activate the intrinsic apoptotic pathway, as shown in in vitro NP/AF cell models and animal studies. These stimuli alter the balance between pro-apoptotic (e.g., Bax, Bak) and antiapoptotic proteins (e.g., Bcl-2, Bcl-xL), leading to mitochondrial outer membrane permeabilization (MOMP) and apoptotic initiation [50].

#### 5.1.1. Mitochondrial Dysfunction and Cytochrome c Release

Following MOMP, cytochrome c is released into the cytosol, where it binds Apaf-1 and procaspase-9 to form the apoptosome [51]. This complex activates caspase-9 and downstream effector caspases (caspase-3/-7), leading to DNA fragmentation. Evidence from human degenerated disc tissues and in vitro studies confirms reduced mitochondrial membrane potential, ATP depletion, and increased Bax translocation [51]. 

#### 5.1.2. Role of Oxidative Stress

Oxidative stress is the principal driver of mitochondrial apoptosis [52]. ROS exposure in NP cells induces p53 activation, Bax upregulation, and Bcl-2 downregulation. ROS also promotes mitochondrial fragmentation via Drp1 phosphorylation. Antioxidants such as MitoQ and N-acetylcysteine have shown efficacy in restoring mitochondrial function and reducing caspase-3 activation in IVDD models [51,52].

### 5.2. Extrinsic (Death-Receptor-Mediated) Pathway

The extrinsic apoptotic pathway is initiated by extracellular ligand binding to death receptors (the tumor necrosis factor receptor (TNFR) family). This has been demonstrated in disc cell cultures treated with TNF-α, FasL, or TRAIL [50]. 

#### 5.2.1. Formation of the Death-Inducing Signaling Complex (DISC)

Ligand-receptor interactions (e.g., Fas–FasL) recruit the Fas-associated death domain (FADD) and procaspase-8 to form the DISC. Activated caspase-8 cleaves Bid and initiates the mitochondrial cascade [50]. Upregulation of Fas and FasL in degenerated discs has been shown in human tissue samples and rat IVDD models. Fas blockade in NP cells attenuates apoptosis and promotes ECM retention.

#### 5.2.2. Cytokine-Mediated Apoptosis in Disc Cells

Proinflammatory cytokines (TNF-α, IL-1β) upregulate Fas, DR5, and caspases. In vitro experiments confirm NF-κB/MAPK-driven gene expression [51,52]. Chronic cytokine exposure has been shown in disc explants to increase TUNEL-positive apoptotic cells, suggesting an autocrine amplification loop.

### 5.3. ER-Stress-Mediated Apoptosis

ER stress-related apoptosis has been identified in bovine and rat AF/NP explant models and IL-1β-treated human NP cells. The UPR shifts from adaptive to apoptotic signaling during chronic stress [47,53].

#### 5.3.1. UPR Signaling and Apoptotic Switch

- CHOP activation suppresses Bcl-2 and upregulates Bax/Bim, thereby tipping the balance toward apoptosis [19,47].
- Caspase-12 (in rodents) and caspase-4 (in humans) mediate ER-specific apoptosis [47,53].
- The PERK–eIF2α–ATF4–CHOP axis links ER and mitochondrial apoptosis [6,53].

#### 5.3.2. ER Stress in Degenerative Discs

Elevated CHOP, glucose-regulated protein 78 (GRP78), activating transcription factor 6 (ATF6), and caspase-12 levels have been detected in human degenerated discs and oxidative stress-induced disc cultures [46,52,53,54,55,56]. ER stress correlates with mitochondrial fragmentation [47]. Chemical chaperone studies (e.g., tauroursodeoxycholic acid (TUDCA) and 4-phenylbutyric acid (4-PBA)) show apoptosis reduction in disc cell cultures [47,52,53].

### 5.4. Autophagy-Associated Apoptosis

While autophagy is generally considered a cytoprotective mechanism that helps maintain cellular homeostasis, emerging evidence from both in vitro and in vivo models of IVDD suggests that under chronic or dysregulated stress, autophagy may transition into a pro-apoptotic process [57,58,59,60]. This shift is particularly evident in conditions involving sustained oxidative stress, nutrient deprivation, or inflammatory stimulation, all characteristic features of the degenerative disc microenvironment.

#### 5.4.1. Dual Role of Autophagy

- In the early stages, autophagy is upregulated via markers such as LC3-II, Beclin-1, and ATG5, which collectively help remove damaged organelles, reduce ROS accumulation, and suppress proinflammatory pathways. This cytoprotective phase is crucial for maintaining ECM integrity and delaying disc degeneration [57,58].
- However, under prolonged or excessive stress, autophagy becomes dysregulated, impairing autophagosome clearance, accumulation, and eventual activation of apoptotic cascades, including caspase-3 and caspase-9 [59,60].
- Pharmacological studies in NP cells have shown that autophagy inhibitors like 3-MA and chloroquine exacerbate apoptosis and ECM degradation, while autophagy activators such as rapamycin and metformin mitigate cell death and support matrix preservation, suggesting a potential therapeutic window for modulating autophagy in IVDD [58,60].

#### 5.4.2. Crosstalk Between Autophagy and Apoptosis

Autophagy and apoptosis are closely interconnected, sharing multiple regulatory molecules and signaling pathways that govern cell fate decisions [61,62]:

- Caspase-3-mediated cleavage of Beclin-1, a central autophagy initiator, inhibits autophagy and concurrently enhances apoptotic signaling, representing a key molecular switch from survival to death [61].
- Accumulation of p62/sequestosome-1, a selective autophagy substrate, can activate the NLRP3 inflammasome, amplifying inflammation and apoptosis under chronic stress [61].
- Autophagy and apoptosis also intersect through the Bcl-2–Beclin-1 complex and p53 signaling, which regulate both mitochondrial stability and lysosomal activity, further coordinating the balance between autophagy and apoptosis [62].

These mechanisms have been validated primarily in in vitro NP cell cultures exposed to inflammatory cytokines and oxidative stress, highlighting the importance of context-dependent regulation in determining whether autophagy is a protective or detrimental process during IVDD progression [61,62].

## 6. Hyperglycemia and Diabetes-Related Apoptosis in Disc Cells

Chronic hyperglycemia is a well-established contributor to IVDD, acting through AGE-RAGE signaling, oxidative stress, mitochondrial dysfunction, and inflammatory cascades [3,11]. Notably, in vitro studies using rat NP and AF cells have shown that high glucose promotes excessive ROS production and mitochondrial dysfunction, leading to oxidative damage and redox imbalance [17,63]. These effects upregulate pro-apoptotic Bax, promote mitochondrial cytochrome c release, and activate caspase-3 and PARP cleavage [3,17,63]. Additionally, in vitro experiments suggest that hyperglycemia suppresses autophagy by inhibiting AMPK phosphorylation and upregulating mTOR signaling, impairing protective cellular mechanisms [64]. In vivo rat disc models have confirmed that high-glucose environments exacerbate disc cell apoptosis through ROS-mediated mitochondrial damage and increased Bax/Bcl-2 ratios [13,63]. AGEs activate RAGE receptors on NP and AF cells, initiating NF-κB, JAK/STAT, and MAPK signaling cascades that amplify inflammation, apoptosis, and matrix degradation [3,11]. These findings, derived mainly from animal and in vitro models, support the concept that metabolic syndrome functions as a modifiable risk factor for IVDD, though longitudinal clinical evidence remains limited [11]. 

### 6.1. AGEs and RAGE Activation

AGEs are a heterogeneous group of molecules formed through the nonenzymatic glycation of proteins, lipids, and nucleic acids under conditions of prolonged hyperglycemia.

In the avascular environment of the intervertebral disc, where glucose diffuses readily but clearance is limited, AGEs accumulate over time and induce structural and functional damage to disc tissues [3,11].

#### 6.1.1. AGE-RAGE Signaling Cascade

AGEs interact with RAGE, activating NF-κB, MAPK (ERK, JNK, p38), and JAK/STAT signaling pathways. These induce proinflammatory cytokines and apoptotic mediators, including Bax and caspase-3. In vitro studies using human NP cells treated with AGE-modified BSA demonstrated increased caspase-3 activity and MMP expression [3,11].

#### 6.1.2. Impact on Disc Cells

AGE accumulation disrupts mitochondrial membrane potential, induces cytochrome c release, and promotes ECM degradation. Bovine NP cell studies confirmed mitochondrial fragmentation and increased oxidative stress upon AGE exposure [17,63].

### 6.2. Oxidative Stress and Mitochondrial Dysfunction

In hyperglycemic disc cells, excessive ROS production results from increased mitochondrial electron transport chain activity and NADPH oxidase. This leads to DNA damage, Bax upregulation, and caspase activation. Diabetic rat disc models revealed increased Bax/Bcl-2 ratios and histological disc degeneration [5,6,19]. 

#### 6.2.1. ROS-Induced Apoptotic Markers

In vitro studies have revealed that elevated ROS levels in diabetic disc cells promote DNA damage, lipid peroxidation, and protein oxidation. These events lead to mitochondrial membrane permeabilization, cytochrome c release, and the activation of the caspase-dependent apoptotic pathway [64,65]. in diabetic NP cells from rat models, pro-apoptotic markers such as Bax, cleaved caspase-3, and PARP are upregulated, while Bcl-2 expression is suppressed. This shift increases the Bax/Bcl-2 ratio, activating the mitochondrial apoptotic cascade and contributing to histological disc degeneration. These findings have been consistently reported in animal studies modeling diabetic disc degeneration [17,66].

#### 6.2.2. Antioxidant Impairment

Hyperglycemia suppresses endogenous antioxidant defenses by reducing the activity of enzymes, including SOD, catalase, and GPx. Studies in diabetic rat disc tissues have shown reduced Nrf2 expression, further amplifying oxidative damage. This loss of redox homeostasis triggers apoptosis and enhances inflammatory cytokine expression, linking oxidative stress to inflammation and ECM catabolism [66,67].

### 6.3. Inflammation in the Diabetic Disc Microenvironment

Proinflammatory cytokines, upregulated in diabetic disc models, contribute to disc degeneration. Elevated levels of TNF-α, IL-1β, IL-6, and monocyte chemoattractant protein-1 (MCP-1) have been detected in disc tissues from rodent models of type 2 diabetes mellitus [11,68].

#### Cytokine-Driven Apoptosis

These cytokines activate both extrinsic and intrinsic apoptotic pathways. In vitro studies have shown that IL-1β downregulates Bcl-2 and upregulates Bax, sensitizing cells to mitochondrial apoptosis. Combined with elevated ROS, this promotes cytochrome c release [68]. Evidence from diabetic mouse models further demonstrates that blocking IL-1β reduces apoptosis and restores ECM expression, suggesting therapeutic potential for cytokine inhibitors [11].

### 6.4. Epigenetic and Transcriptional Regulation

Several microRNAs (miRNAs) are dysregulated in diabetic intervertebral discs. Among them, miR-34a and miR-155 are upregulated and promote apoptosis by targeting antiapoptotic genes such as Bcl-2 and sirtuin 1 (SIRT1) [69]. In contrast, miR-21—known for its antiapoptotic and anti-inflammatory properties—is frequently downregulated under diabetic conditions. Therapeutic delivery of miR-21 mimics or inhibition of miR-34a has been shown to reduce apoptosis and restore mitochondrial function in NP cells exposed to hyperglycemic stress [70,71].

#### 6.4.1. miRNA Dysregulation

Several miRNAs are dysregulated in diabetic discs. In vitro studies have shown that miR-34a and miR-155 are upregulated, promoting apoptosis by targeting Bcl-2 and SIRT1, while miR-21 is downregulated under hyperglycemic stress. Therapeutic delivery of miR-21 mimics in diabetic NP cells has been shown to restore mitochondrial function and reduce apoptosis [70,71].

#### 6.4.2. SIRT1 Downregulation

SIRT1 protects against oxidative stress and apoptosis. Hyperglycemia reduces SIRT1 expression, increasing p53 acetylation and promoting apoptotic gene transcription. In vitro activation of SIRT1 by agents such as resveratrol has been shown to restore redox homeostasis in diabetic NP cells [70,71]. 

Research shows that hyperglycemia causes IVDD through various apoptotic mechanisms, such as AGE-RAGE signaling, ROS generation, ER stress, inflammation, and miRNA pathways. Targeted therapies such as AGE inhibitors, antioxidants, and SIRT1 activators warrant further clinical exploration [71]. 

## 7. Inflammation-Induced Apoptosis in IVDD

Multiple in vitro and in vivo studies have demonstrated that proinflammatory cytokines—particularly TNF-α and IL-1β—are upregulated in degenerative discs and contribute to disc cell apoptosis by activating the extrinsic death receptor pathway [36,72,73]. The binding of TNF-α to TNFR1 or Fas ligand to Fas receptor leads to caspase-8 cleavage and Bid-mediated mitochondrial disruption [72]. In addition, inflammatory stress promotes ER stress in NP cells, as evidenced by elevated expression of CHOP and GRP78 [36,74]. NF-κB signaling, extensively investigated in both disc cell and animal models, is a central mediator of inflammation that regulates both prosurvival and pro-apoptotic genes. However, chronic NF-κB activation skews this balance toward apoptosis, particularly under persistent inflammatory conditions [36,73]. Importantly, inflammation-induced apoptosis and ECM degradation reinforce each other, creating a deleterious degenerative feedback loop [72]. Notably, inflammation-induced apoptosis and ECM degradation are closely intertwined, creating a degenerative feedback loop (Table 2).

### 7.1. Proinflammatory Cytokines in Degenerative Discs

Elevated TNF-α, IL-1β, IL-6, and IL-17 levels have been consistently observed in human degenerative disc tissues and in animal models of IVDD [72,75,76]. These cytokines are secreted by NP and AF cells and activated immune cells, including macrophages and Th17 lymphocytes. Their expression is triggered by mechanical overloading, oxidative stress, and ECM degradation products. Once released, these cytokines upregulate matrix-degrading enzymes such as MMP-3 and MMP-13 while downregulating anabolic matrix components like aggrecan and type II collagen, ultimately promoting apoptosis [72,73]. 

#### 7.1.1. Cytokine-Mediated Signaling Pathways

TNF-α binding to TNFR1 activates IκB kinase (IKK) complexes, leading to NF-κB nuclear translocation and subsequent upregulation of pro-apoptotic genes such as Bax and Fas, as well as inflammatory mediators like COX-2 and inducible nitric oxide synthase (iNOS) [36,73]. IL-1β activates both NF-κB and MAPK signaling cascades—including ERK, p38, and JNK—via IL-1R1, which promotes caspase-3 expression and mitochondrial outer membrane permeabilization (MOMP) leading to caspase-9 activation [72,74]. In vitro studies show that blocking TNF-α or Fas signaling reduces the number of TUNEL-positive apoptotic NP cells, highlighting their direct role in apoptosis initiation [72].

#### 7.1.2. Amplification of Apoptotic Signaling

Interactions with oxidative and ER stress further potentiate cytokine-driven apoptotic pathways. IL-1β downregulates the antiapoptotic protein Bcl-2 while upregulating Bax, shifting the mitochondrial membrane potential toward apoptosis [74,75]. Elevated ROS levels, observed in cytokine-stimulated NP cells, exacerbate mitochondrial dysfunction. Simultaneously, inflammatory cytokines increase ER stress via upregulating CHOP, GRP78, and activating transcription factor 4 (ATF4), leading to caspase-12 activation [73]. Rodent studies confirm that simultaneous exposure to cytokines and oxidative stress produces synergistically elevated caspase-3 and -9 activity compared to individual stimuli [74]. 

### 7.2. Role of IL-17 and Th17 Cells

IL-17, primarily secreted by Th17 cells, has emerged as a critical cytokine in disc degeneration and apoptosis. Studies in both human samples and animal models have shown a strong correlation between IL-17 levels and IVDD severity [76]. IL-17 amplifies the inflammatory milieu by increasing TNF-α and IL-1β production in disc cells and by activating NF-κB and C/EBPβ signaling pathways [73,77]. This cascade enhances matrix-degrading enzyme expression and upregulates Fas and caspase-3. In vitro, IL-17 increases ROS and cleaved caspase-3 levels in NP cells, whereas in vivo treatment with IL-17-neutralizing antibodies reduces inflammation, preserved disc height, and attenuates apoptosis [76].

### 7.3. Toll-like Receptors and Innate Immune Activation

Toll-like receptors (TLRs), particularly TLR2 and TLR4, are expressed in human and rat disc cells and recognize damage-associated molecular patterns (DAMPs), such as degraded ECM fragments and heat shock proteins [36]. TLR activation triggers MyD88-dependent NF-κB and p38 MAPK pathways, leading to upregulating Fas ligand (FasL), TNF-related apoptosis-inducing ligand (TRAIL), and cytochrome c release. Silencing TLR4 using small interfering RNA (siRNA) in NP cells has been shown to reduce proinflammatory cytokine expression and apoptosis, while in vivo inhibition of TLR4 attenuates disc degeneration in rodent models [36].

### 7.4. Crosstalk with Oxidative and ER Stress

There is a well-established crosstalk between inflammatory cytokines, oxidative stress, and ER stress in IVDD. Cytokines enhance mitochondrial ROS generation by activating redox-sensitive transcription factors such as NF-κB and AP-1, which further upregulate cytokine gene expression, creating a positive feedback loop [36,73]. Concurrently, ER stress is intensified due to excessive protein synthesis and misfolded protein accumulation. The unfolded protein response (UPR), particularly via the PERK–eIF2α–CHOP axis, upregulates pro-apoptotic proteins such as Bim and death receptor 5 (DR5), increasing apoptosis susceptibility. Co-treatment of NP cells with IL-1β and hydrogen peroxide (H_2_O_2_) induces synergistic activation of caspase-12, -9, and -3, confirming the additive pro-apoptotic effects of inflammatory and oxidative stimuli [74]. 

## 8. Therapeutic Strategies Targeting Apoptosis in IVDD

**Figure 2:**
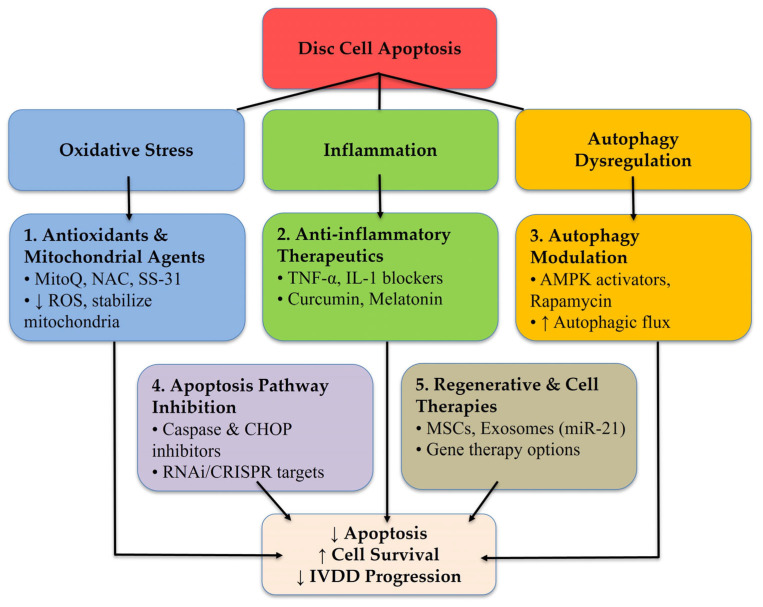
Therapeutic strategies to mitigate disc cell apoptosis in intervertebral disc degeneration (IVDD). The figure summarizes current therapeutic approaches aimed at reducing apoptosis of disc cells, including NP and AF cells. These strategies are categorized into five main domains: (1) antioxidants and mitochondrial-targeted agents to reduce oxidative stress and preserve mitochondrial function [79,80]; (2) anti-inflammatory therapeutics targeting proinflammatory cytokines and signaling pathways [76,77,78,79,81]; (3) autophagy modulators to enhance cytoprotective autophagic flux [79,80,82]; (4) inhibitors of apoptotic signaling pathways such as caspases, Bcl-2 family proteins, and ER stress mediators [76,77]; and (5) regenerative and cell-based therapies including mesenchymal stem cell transplantation [78,83,84,85], exosome-based delivery of antiapoptotic microRNAs [86,87,88,89,90], and gene therapy approaches [90,91]. These interventions act at different levels of the apoptotic cascade and promise to halt or reverse disc degeneration.

Given the central role of apoptosis in IVDD pathogenesis, targeting apoptotic pathways offers promising therapeutic strategies to preserve disc cell viability and function. Various pharmacological, genetic, and biological interventions have been explored in preclinical settings (Figure 2) [76,77]. However, limitations remain, such as poor bioavailability, challenges in ECM penetration, immunogenicity, and delivery issues. Current evidence is preclinical; human trial data remain scarce and often limited in scale. These include:

- Anti-inflammatory agents (e.g., curcumin, IL-1Ra) suppress cytokine-driven apoptosis via NF-κB and MAPK inhibition [76,77,78,79].
- Mitochondrial antioxidants (e.g., MitoQ) reduce ROS and stabilize mitochondrial function [79,80,81].
- Caspase inhibitors (e.g., Z-VAD-FMK) block downstream apoptotic signaling [76,77].
- Autophagy modulators (e.g., rapamycin) promote cellular homeostasis [79,80,82].
- Biological approaches using mesenchymal stem cells (MSCs) [78,83,84,85] and exosomes [86,87,88,89,90] offer antiapoptotic and regenerative effects.
- Gene therapies (e.g., siRNA, miRNA, CRISPR–Cas9) modulate apoptosis-related genes [90,91].

Although promising, these therapies face limited bioavailability, delivery into avascular discs, and immunogenicity. Current evidence remains mostly preclinical, with few clinical trials available.

### 8.1. Antioxidants and Mitochondrial Protectants

Antioxidants have demonstrated antiapoptotic effects in IVDD by reducing oxidative stress and preserving mitochondrial function. In vitro and in vivo studies using NP and AF cells have shown that agents like N-acetylcysteine, resveratrol, melatonin, and mitochondrial protectants (e.g., MitoQ, CoQ10) reduce ROS, prevent apoptosis, and preserve disc structure [79,80]. 

#### 8.1.1. Mitochondrial-Targeted Antioxidants

Mitochondria are the primary source and key target of oxidative damage in intervertebral disc cells. Mitochondria-targeted agents such as MitoQ, SkQ1, and SS-31 have demonstrated superior efficacy in preserving mitochondrial membrane potential, preventing cytochrome c release, and activating SIRT3, PGC-1α, and FOXO3a, thus enhancing mitochondrial resilience [79,80].

#### 8.1.2. Natural Antioxidants and Polyphenols

Natural compounds, including resveratrol, curcumin, and epigallocatechin-3-gallate (EGCG), exert antioxidant, anti-inflammatory, and antiapoptotic effects via multiple pathways relevant to IVDD [79].

- Resveratrol: Resveratrol activates the SIRT1/Nrf2 pathway, enhancing cellular antioxidant defenses. It also promotes autophagy and supports ECM synthesis by upregulating genes responsible for type II collagen and aggrecan production. Resveratrol further reduces pro-apoptotic signaling by inhibiting caspase activity and stabilizing mitochondrial function [79].
- Curcumin: Curcumin induces heme oxygenase-1 (HO-1) and NAD(P)H quinone dehydrogenase 1 (NQO1), both downstream targets of Nrf2 activation. It also inhibits NF-κB signaling, suppressing inflammatory cytokine production and MMP expression in disc cells. These combined effects help preserve disc integrity under stress conditions [80].
- EGCG: EGCG inhibits H_2_O_2_-induced apoptosis by modulating PI3K/Akt and MAPK signaling pathways. It also promotes mitochondrial stability, reduces ROS accumulation, and enhances the expression of antiapoptotic proteins in NP cells subjected to oxidative stress [79].

#### 8.1.3. Therapeutic Potential and Challenges

Despite strong preclinical results, antioxidants suffer from low disc bioavailability and short half-lives. To improve their targeting efficiency, emerging delivery strategies, including ROS-responsive nanoparticles and hydrogels, are being explored [77,80]. 

### 8.2. Anti-Inflammatory Agents and Cytokine Inhibitors

Cytokines such as TNF-α, IL-1β, IL-6, and IL-17 are central to inflammation-driven apoptosis in IVDD. Inhibitors of these cytokines significantly reduce disc degeneration and ECM breakdown in animal models [78,79].

#### 8.2.1. Cytokine Inhibitors

- Etanercept (TNF-α blocker): A soluble TNF receptor fusion protein, etanercept effectively neutralizes TNF-α signaling. It has been shown to reduce apoptosis, suppress inflammatory cascades, and improve histological preservation of disc architecture in rodent herniation and degeneration models [78].
- Anakinra (IL-1Ra): This recombinant IL-1 receptor antagonist inhibits IL-1β activity, reducing MMP expression, decreasing ROS production, and enhancing type II collagen synthesis. Anakinra-treated disc cells exhibit improved redox balance and ECM maintenance in oxidative stress conditions [79].
- Tocilizumab (anti–IL—6R) and Secukinumab (anti–IL—17A): These monoclonal antibodies target interleukin signaling and have shown encouraging anti-inflammatory and antiapoptotic effects in animal models of IVDD. Tocilizumab modulates IL–6–mediated catabolic responses, while Secukinumab reduces IL–17–driven apoptosis and inflammation [76,78].

These cytokine inhibitors highlight the therapeutic potential of targeting specific inflammatory mediators in disc degeneration, although clinical translation requires further validation regarding safety, delivery, and long-term efficacy [77,81].

#### 8.2.2. NF-κB Pathway Inhibitors

The NF-κB signaling pathway is a central regulator of inflammation, apoptosis, and catabolic enzyme expression in disc degeneration. Small-molecule inhibitors such as BAY 11-7082, parthenolide, curcumin, and celastrol have demonstrated significant efficacy in suppressing NF-κB activation in both NP and AF cells [80,82]. These agents reduce the transcription of inflammatory cytokines, matrix-degrading enzymes (e.g., MMPs), and pro-apoptotic mediators.

#### 8.2.3. Future Perspectives and Combination Strategies

Precision strategies combining anti-inflammatory agents with antioxidants or autophagy inducers (e.g., rapamycin) are developing actively [77].

### 8.3. Autophagy Regulators

Autophagy is a key cytoprotective process that maintains cellular homeostasis by clearing damaged organelles and proteins. In the context of IVDD, autophagy activation in NP cells helps mitigate oxidative and inflammatory stress, thereby preserving mitochondrial function and reducing apoptosis. Pharmacological agents such as rapamycin and metformin have demonstrated robust effects in restoring mitochondrial membrane potential, enhancing autophagic flux, and suppressing caspase activation in various in vitro and in vivo disc degeneration models [79,82].

#### 8.3.1. Autophagy-Promoting Agents

Several compounds directly stimulate autophagy and have shown protective effects in IVDD:

- Rapamycin: A classical mTORC1 inhibitor, rapamycin promotes mitophagy, the selective autophagic removal of damaged mitochondria. In IVDD models, it improves disc cell viability, reduces apoptosis, and enhances matrix synthesis by restoring mitochondrial quality control [82].
- Metformin: Widely used in diabetes treatment, metformin activates the AMPK pathway, reduces CHOP and caspase-3 expression, and upregulates autophagy markers, including Beclin-1 and LC3-II. Its ability to regulate energy metabolism and stress responses makes it a promising agent in IVDD therapy [79].
- Resveratrol and trehalose: Both compounds support autophagic flux through distinct mechanisms. Resveratrol enhances SIRT1/AMPK signaling, while trehalose acts independently of mTOR. They contribute to ECM preservation and reduce oxidative injury in NP cells [79].

#### 8.3.2. Dual-Function Compounds

Certain naturally derived compounds exert both autophagy-inducing and anti-inflammatory effects, making them particularly valuable for multifactorial disorders like IVDD:

- Melatonin: Beyond its antioxidant activity, melatonin activates AMPK/SIRT1 signaling, reduces intracellular ROS, and promotes the synthesis of type II collagen and aggrecan. These effects contribute to improved ECM homeostasis and reduced degeneration [79].
- Berberine: This isoquinoline alkaloid modulates the AMPK–Beclin-1 axis, enhancing autophagy while simultaneously suppressing TNF-α and IL-1β expression. Berberine has reduced apoptosis and ECM breakdown in disc cell models [79].
- Curcumin: Known for its anti-inflammatory and antioxidant properties, it modulates the PI3K/Akt/mTOR pathway, promoting autophagy under stress conditions. It helps counteract inflammation-induced apoptosis and supports disc cell survival [80].

#### 8.3.3. Challenges and Future Perspectives

Delivery issues and precise dose optimization remain significant barriers. ROS- or pH-responsive nanocarriers are being developed [77,82].

### 8.4. Stem Cell and Exosome-Based Therapies

Stem cell-based and exosome-based approaches promise to reverse disc degeneration by modulating inflammation, apoptosis, and ECM repair. While preclinical results are encouraging, issues remain regarding cell survival, integration, and long-term safety.

#### 8.4.1. MSC Transplantation

MSCs improve disc structure by secreting paracrine factors like TGF-β, stromal-cell-derived factor-1 (SDF-1), HGF, and IGF-1 that suppress apoptosis and promote ECM synthesis [78,83,84]. Hydrogel encapsulation improves their mechanical stability and survival [83,85].

#### 8.4.2. Exosome Therapy

Exosomes are nanosized extracellular vesicles (30–150 nm in diameter) secreted by MSCs that contain lipids, proteins, and nucleic acids, including miRNAs and long non-coding RNAs (lncRNAs). These vesicles mediate many MSCs’ therapeutic effects by facilitating intercellular communication and delivering regulatory molecules to recipient disc cells [81,82,83,84]. MSC-derived exosomes contain miRNAs (e.g., miR-21, miR-146a, miR-532-5p) that suppress phosphatase and tensin homolog (PTEN), Bax, NF-κB, and MAPK signaling, thereby reducing inflammation and apoptosis [86,87,88,89].

#### 8.4.3. Challenges and Future Directions

Limitations include delivery, standardization, and manufacturing hurdles. Future approaches include exosome engineering, pre-loading with miRNAs, and the use of responsive scaffolds [87,88,89]. 

### 8.5. Gene Therapy and Molecular Targeting

Gene therapy presents a promising strategy for IVDD by modulating apoptosis-related genes. Approaches such as siRNA, miRNA, and CRISPR/Cas9 have demonstrated efficacy in preclinical models; however, delivery, specificity, and safety challenges persist [90,91].

#### 8.5.1. siRNA and miRNA-Based Therapies

siRNA and miRNA represent powerful tools for post-transcriptional gene regulation. siRNAs achieve gene silencing by guiding the RNA-induced silencing complex (RISC) to specifically degrade target mRNA transcripts, whereas miRNAs fine-tune gene expression primarily by inhibiting translation or promoting mRNA degradation.

siRNAs have been employed to silence critical pro-apoptotic genes, including Bax, caspase-3, and Fas, thereby reducing apoptosis in NP cells. For instance, siRNA-mediated knockdown of Bax significantly attenuates mitochondrial membrane permeabilization and caspase-9 activation in oxidative stress models. Similarly, targeted delivery of caspase-3 siRNA preserves ECM synthesis and prevents cell death under inflammatory conditions [90].

miRNAs serve as endogenous regulators of cell fate and are frequently dysregulated in degenerative disc tissue. Therapeutic delivery of miRNAs such as miR-21, miR-146a, miR-223, and miR-30d has demonstrated promising results. For example, miR-21 upregulates the antiapoptotic protein Bcl-2 and suppresses PTEN, enhancing cell survival. miR-146a targets tumor necrosis factor receptor-associated factor 6 (TRAF6) and interleukin-1 receptor-associated kinase 1 (IRAK1), pivotal mediators of the NF-κB inflammatory pathway, thereby reducing cytokine-induced apoptosis [79,85]. Additionally, certain miRNAs indirectly modulate autophagy and ER stress, exerting broader cytoprotective effects.

However, poor stability and uptake limit RNA delivery into the avascular disc. Nanoparticles, polymers, and exosome-based vectors are under development to improve targeting and bioavailability.

#### 8.5.2. Gene Editing and Vector-Based Delivery

The clustered regularly interspaced short palindromic repeats-associated protein 9 (CRISPR/Cas9) enables direct editing of genes regulating apoptosis (e.g., *caspase-8*, *SIRT1*), with favorable effects on mitochondrial function and ECM integrity in animal models. Viral vectors (e.g., adeno-associated virus (AAV), lentivirus) offer efficient delivery but carry risks such as immunogenicity and insertional mutagenesis. Non-viral vectors (e.g., polyethyleneimine (PEI), hydrogels) are safer but less efficient. Hybrid or physical delivery methods (e.g., ultrasound, electroporation) are being explored to optimize safety and efficacy [90,91].

#### 8.5.3. Clinical Potential and Challenges

Key issues include off-target effects, safety, and low penetration into avascular disc tissue. Combining gene therapy with MSCs or exosomes may overcome these hurdles [91].

### 8.6. Preclinical or Clinical Investigations Targeting Disc Cell Apoptosis in IVDD

Table 3 provides an overview of emerging therapies currently under preclinical or clinical investigations that aim to inhibit disc cell apoptosis and mitigate IVDD.

## 9. Conclusions and Future Directions

Apoptosis of disc cells is a key contributor to IVDD and associated low back pain. It is driven by mitochondrial dysfunction, death receptor pathways, ER stress, and autophagy, influenced by hypoxia, mechanical stress, hyperglycemia, oxidative stress, and inflammation.

Preclinical strategies—including antioxidants, cytokine inhibitors, autophagy enhancers, gene therapy, and exosome-based approaches—potentially mitigate apoptosis and preserve disc integrity. Among these, targeting mitochondrial and inflammatory pathways appears especially promising. However, clinical translation remains difficult due to poor vascularization, limited drug penetration, and the multifactorial nature of IVDD. Although this review integrates extensive preclinical evidence, many cited mechanisms require validation in human tissues and clinical settings. The lack of high-level prospective human data remains a significant limitation of current understanding. Moreover, the translational gap between molecular insights and therapeutic application persists.

Future therapies will likely require multimodal, personalized approaches supported by (a) Improved delivery systems. (b) Biomarkers for early detection and treatment response, and (c) Optimized dosing regimens. Advancing our understanding of disc cell apoptosis will be critical to shifting from symptom relief to disease-modifying therapies, ultimately restoring disc function and improving patient outcomes.

## Figures and Tables

**Table 1:** The pathophysiological mechanism of intervertebral disc degeneration (IVDD).

Process	Key Molecular Mechanism	Consequences
1. Disc Dehydration [1,3]	↓ Aggrecan and GAGs (MMP-3, -13, ADAMTS-4/5) ↑ Type I collagen (fibrosis)	↓ Disc height and turgor Endplate fractures Facet degeneration
2. ECM Breakdown [1,3]	↑ MMP-1, -3, -13, ADAMTS-5 ↓ TGF-β, IGF-1, BMP-7 ECM → DAMPs	↓ Elasticity AF tearing CEPs calcification
3. Inflammation [1,3,43]	↑ TNF-α, IL-1β, IL-17, MCP-1 NF-κB, MAPKs, JAK/STAT activation ↑ NGF, BDNF	↑ Pain sensitization ↓ Matrix synthesis ↑ Apoptosis
4. Oxidative Stress [5,6,17,40,41,42]	↑ ROS (mitochondria, NADPH oxidase) ↓ SOD, Catalase, Nrf2 Redox-sensitive TFs (NF-κB, AP-1)	Mitochondrial dysfunction Cell senescence Inflammation loop
5. Apoptosis [6,7,20,44]	Intrinsic: Bax, Caspase-9/-3 ER stress: CHOP, Caspase-12 Extrinsic: Fas, DR5	NP/AF cell death ↓ ECM synthesis Structural collapse
6. Necrosis/Necroptosis [45]	RIPK1, RIPK3, MLKL DAMP release → TLRs Inflammation amplification	Persistent degeneration Chronic pain Immune activation

**Table 2:** Key proinflammatory mediators in intervertebral disc degeneration (IVDD) and their apoptotic effects.

Cytokine	Effects of Apoptosis	Mechanisms Involved
TNF-α [72,73,74]	Activates extrinsic (death receptor) and intrinsic (mitochondrial) apoptotic pathways Induces mitochondrial dysfunction and ROS Production Increases MMP and ADAMTS expression Promotes ECM breakdown	Death receptor signaling (TNFR1, FADD, Caspae-8) Mitochondrial dysfunction (Cytochrome c release, Caspase-9 activation) Oxidative-stress-mediated apoptosis
IL-1β [36,73,75]	Suppresses ECM synthesis while enhancing catabolic enzyme production Activates NF-κB and MAPK pathways Amplify inflammatory and apoptotic Responses	NF-κB and MAPK activation Suppression of ECM synthesis Induction of pro-apoptotic gene expression
IL-6 [75]	Promotes chronic inflammation and sensitizes disc cells to TNF-α-induced Apoptosis	Enhancement of TNF-α apoptotic effects Prolonged inflammatory responses
IL-17 [76]	Exacerbates apoptosis through STAT3 activation, mainly secreted by infiltrating Immune cells	STAT3-mediated apoptosis signaling Immune cell infiltration promoting apoptotic cascades

**Table 3:** Summary of preclinical or clinical investigations targeting disc cell apoptosis in IVDD.

Therapeutic Strategy	Mechanism of Action	Evidence Type	Key References
MitoQ (Mitoquinone)	Mitochondrial antioxidant, reduces ROS	Rodent IVDD model	Bao et al., 2023 [4]
N-acetylcysteine (NAC)	Redox balance restoration, antioxidant	In vitro NP/AF cell culture	Yurube et al., 2023 [20]
Rapamycin	Induces autophagy via mTOR inhibition	Rodent IVDD model	Yurube et al., 2024 [13]
Metformin	Enhances autophagy, Inhibits apoptosis via AMPK	In vivo rat NP model	Chen et al., 2016 [79]
Etanercept (TNF-α blocker)	Inhibits TNF-α-mediated apoptosis and inflammation	Rat herniation model	Onda et al., 2004 [8]
Anakinra (IL-1Ra)	Suppresses IL-1β signaling, Reduces MMPs and ROS	In vitro & in vivo disc models	Dong et al., 2024 [9]
MSC-derived Exosomes	Delivers antiapoptotic miRNAs, support ECM	In vivo rat IVDD model	Liao et al., 2019 [14]
miRNA therapy (miR-21)	Regulates apoptotic gene expression (e.g., Bcl-2, PTEN)	In vitro NP cell model	Xi et al., 2017 [15]

## Data Availability

Not applicable.

## Abbreviations

The following abbreviations are used in this manuscript:

IVDD	Intervertebral Disc Degeneration
IVD	Intervertebral Disc
NP	Nucleus Pulposus
AF	Annulus Fibrosus
CEP	Cartilaginous Endplates
ECM	Extracellular Matrix
ER	Endoplasmic Reticulum
TNF-α	Tumor Necrosis Factor-alpha
IL-1β	Interleukin-1 beta
MMP	Matrix Metalloproteinases
AIF	Apoptosis-Inducing Factor
ROS	Reactive Oxygen Species
TNF-α	Tumor Necrosis Factor-alpha Clues Pulposus
IL-1β	Interleukin-1 beta
FasL	Fas Ligand
MSCs	Mesenchymal Stem Cells
NCs	Notochordal Cells
TGF-β	Transforming Growth Factor-beta
BMPs	Bone Morphogenic Proteins
IGF-1	Insulin-like Growth Factor-1
